# *Bacillus* spp. supplementation promotes feed efficiency in mid- to late-lactation dairy cows and affects rumen fermentation traits of rumen-fistulated females offered a corn silage–based total mixed ration diet

**DOI:** 10.3168/jdsc.2024-0686

**Published:** 2025-02-20

**Authors:** Bruno A.V. Arthur, Luiz Gustavo Nussio, Oscar C.M. Queiroz, Greicieli de Morais, Rafael H.P. Reis, Giuseppe Copani, Jens N. Joergensen, Bruno I. Cappellozza

**Affiliations:** 1 Department of Animal Science, College of Agriculture “Luiz de Queiroz,” University of São Paulo, Piracicaba, SP, 13418-900, Brazil; 2 Novonesis, Lyngby 2970, Denmark; 3 Federal Institute of Education, Science and Technology of Rondônia, Colorado do Oeste 76993-000, RO, Brazil

## Abstract

Two experiments evaluated the effects of supplementing a *Bacillus*-based direct-fed microbial (DFM) on productive performance of mid- to late-lactating dairy cows (experiment [Exp.] 1), and on rumen fermentation traits of rumen-fistulated Holstein cows (Exp. 2). In Exp. 1, 60 mid-lactating (126 ± 11.5 DIM), primiparous (n = 14), and multiparous (n = 46) Holstein cows were blocked, within parity, by milk yield into (1) TMR (CON; n = 30) and (2) CON with *Bacillus licheniformis* 809 and *Bacillus subtilis* 810 (n = 30; BAC). Diets were offered for 12 wk, following a 3-wk covariate period. All cows received a corn silage–based TMR throughout the study. Dry matter intake and milk yield were evaluated daily, whereas milk, blood, and fecal samples were collected on wk 4, 8 and 12 for milk composition, metabolite analysis (urea and glucose), and nutrient digestibility, respectively. In Exp. 2, 16 rumen-fistulated nonlactating multiparous Holstein cows were ranked by initial BW and assigned to the treatments described in Exp. 1. The experimental period lasted 25 d and samples were taken on d 22 to 25 for in situ DM degradability, rumen ammonia, and pH. All data were analyzed with the MIXED procedure of SAS using cow as the experimental unit. In Exp. 1, DMI was reduced in BAC-fed cows, but no differences were observed on milk yield. Cows offered BAC had a grater feed efficiency versus CON (+100 g/kg feed). *Bacillus* spp. supplementation increased milk fat content, reduced MUN, and tended to reduce milk protein content. Mean BUN was reduced and plasma glucose was greater in cows fed BAC. No treatment effects were observed for DM or starch digestibility, but NDF digestibility was greater for BAC-fed cows. In Exp. 2, a treatment × hour interaction was observed on DM degradability, being greater at 12 h postfeeding in BAC cows versus CON. Rumen pH tended to be greater for BAC at 12, 24, and 72 h, whereas mean rumen pH and DM degradability were greater in BAC. In summary, feeding a *Bacillus*-based DFM to mid- to late-lactating dairy cows improved feed efficiency and increased mean glucose, while also stimulating DM and NDF digestibility.

## Highlights

• Direct-fed microbials are feed additives that support the health of the herd.
• *Bacillus* spp. may also affect nutrient digestibility and rumen fermentation traits.
• Feed efficiency was improved in mid- to late-lactating dairy cows fed *Bacillus* spp.
• Rumen fermentation and nutrient digestibility were also altered by *Bacillus* spp.

## Graphical Abstract

**Figure:**
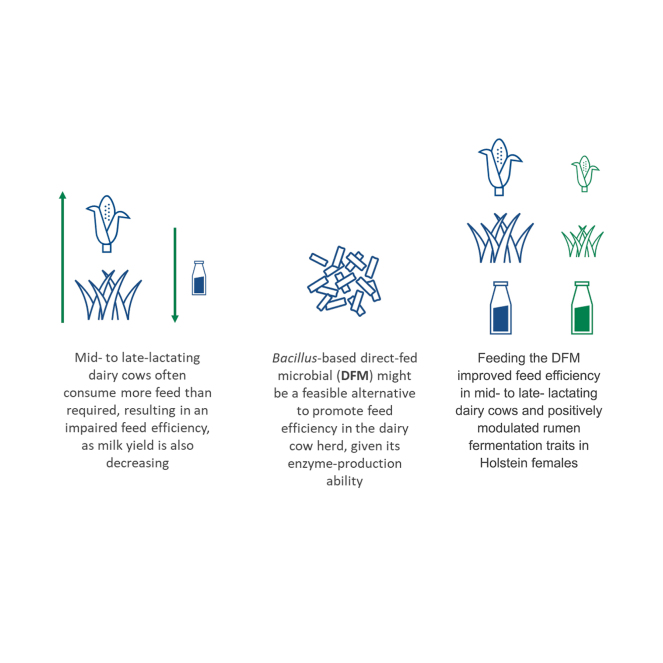
**Summary:** Feed efficiency has been considered one of the key traits affecting the productivity and profitability of dairy operations, as it represents the amount of milk produced per kilogram of feed consumed. In previous studies, feeding *Bacillus licheniformis* 809 and *Bacillus subtilis* 810 improved feed efficiency in early- to mid-lactating dairy cows. Overall, we demonstrated that feed efficiency was improved when a *Bacillus*-based direct-fed microbial (DFM) containing B. licheniformis 809 and B. subtilis 810 was added to a corn silage-based diet and fed to mid- to late-lactating dairy cows. Moreover, nutrient digestibility and rumen fermentation traits were also positively affected following the supplementation with the *Bacillus*-based DFM. In summary, the use of *Bacillus* spp. seems to be a feasible strategy to improve the performance of the dairy cow herd while also optimizing nutrient utilization and fermentation by ruminants.

Mid- to late-lactating dairy cows demonstrate a faster reduction in milk yield than DMI (Van Saun and Sniffen, 1996; Arndt et al., 2015; Ben Abdelkrim et al., 2021), whereas increases in BCS (Mao et al., 2004) and nutrient partitioning (Kirkland and Gordon, 2001; Bach et al., 2020) might be observed. It is often reported that dairy cows in advanced stages of lactation are offered an imbalanced energy, protein, and mineral diet (Garg et al., 2013) that may affect the lactation performance to an even greater extent. Given this scenario, feed efficiency, reported as kilograms of milk per kilogram of feed consumed, might be negatively affected (Bach et al., 2020), which, in turn, affects the profitability of the dairy production system (Thomas et al., 2023). Given that feed is the greatest external factor affecting the profitability of the dairy farm, it is paramount to develop strategies that support milk production of mid- to late-lactating dairy cows, while also improving nutrient utilization and the feed efficiency of the dairy cow herd.

In lactating dairy cattle, daily supplementation of direct-fed microbials (**DFM**) containing or based on *Bacillus* spp. have been shown to improve milk yield (Cappellozza et al., 2024), 3.5% FCM yield (Oyebade et al., 2023), milk component yield (protein, lactose, and fat), and feed efficiency (Cappellozza et al., 2024; Terré et al., 2024) in cows offered a TMR or a partial mixed ration diet. However, most of these studies have been conducted with early- to mid-lactating dairy cows, which may limit the applicability of results when feeding mid- to late-lactating dairy cows. We hypothesize that feeding a bacteria-based DFM will positively affect the feed efficiency of mid- to late-lactating Holstein dairy cows. Therefore, 2 experiments were conducted to evaluate the effects of a *Bacillus*-based DFM on (1) milk production, composition, and feed efficiency of mid- to late-lactating Holstein dairy cows and (2) rumen fermentation traits of rumen-fistulated nonlactating Holstein cows.

Both experiments were conducted at the University of São Paulo, Luiz de Queiroz College of Agriculture, located in Piracicaba, São Paulo, Brazil (22°43′31″S, 47°38′51″W, and elevation of 546 m) from September 2021 to May 2022. All animals were cared for in accordance with the practices outlined in the *Guide for the Care and Use of Agricultural Animals in Research and Teaching* (FASS, 2010) and the studies were approved by the Institutional Ethics Committee for Animal Research of the University of São Paulo (CEUA #2609120221).

Sixty mid-lactation (126 ± 11.5 DIM; 21.7 ± 5.85 kg of milk) primiparous (n = 14) and multiparous (n = 46) Holstein cows were blocked, within parity, by milk yield into 1 of 2 treatments for a 12-wk experimental period: (1) corn silage–based TMR without DFM supplementation (**CON**; n = 30; 7 primiparous and 23 multiparous) or (2) CON with the addition of 3 g/head per day of a *Bacillus*-based DFM containing *Bacillus licheniformis* 809 and *Bacillus subtilis* 810 (**BAC**; 3.2 × 10^9^ cfu/g; BOVACILLUS, Novonesis, Lyngby, Denmark; n = 30; 7 primiparous and 23 multiparous). The basal TMR consisted of 56.1% (DM basis) corn silage and did not contain any other feed additive, such as monensin sodium. Table 1 presents the composition and nutritional profile of the basal TMR. Cows were housed in 6 freestall pens with individual sand beddings that contained 10 individual electronic feeders (Intergado, Contagem, MG, Brazil) per pen and 60 cm of linear bunk space/cow. Cows were milked twice daily (0500 and 1700 h) and diets were offered in equal amounts twice per day (0600 and 1800 h). The particle size distribution of the TMR was measured with the Penn State Particle Separator (**PSPS**) according to the methodology described by Kononoff and Heinrichs (2003).

**Table 1:** Composition and nutritional profile of the basal TMR diet offered in experiments 1 and 2^1^

Item	Value
Ingredient, % DM	
Corn silage	56.1
Ground corn	20.1
Soybean meal	12.4
Citrus pulp	9.0
Mineral-vitamin premix	1.7
Urea	0.7
Nutritional profile, % or % DM	
DM	61.9
NDF	35.9
CP	17.8
Starch	20.6
Ash	1.8
PSPS, % of feed retained^2^	
19-mm	4.13
8-mm	49.94
4-mm	20.30
Bottom pan	25.63

1 Diets were offered twice daily for the entire experimental periods.

2 Measured as described by Kononoff and Heinrichs (2003).

From d −21 to 0, cows were fed the basal TMR for adaptation to the specific bunkers that they would have access to during the experiment, as well as for determination of daily DMI and milk yield to be used as covariates. Throughout the experimental period, DMI, milk yield, and feed efficiency were determined daily, whereas a milk sample was collected twice per week for determination of milk fat, protein, lactose, MUN, and SCC in a commercial laboratory (Clínica do Leite, Universidade de São Paulo, Piracicaba, SP, Brazil). Blood samples were collected on d 47, 75, and 103 of the study at 1, 2, 3, 6, and 12 h post-TMR feeding in the morning for determination of BUN and glucose. Samples were taken via the jugular vein using Vacutainer tubes with sodium fluoride (for glucose) and heparin (for BUN), immediately placed on ice and centrifuged at 3,000 × *g* for 20 min at 4°C until further laboratory analyses. Blood samples were analyzed via enzymatic colorimetric kits for glucose (K082, Bioclin Quibasa, Belo Horizonte, MG, Brazil) and BUN (Urea UV, Bioclin Quibasa), respectively.

Individual fecal samples were collected by manually taking fecal contents (fecal grab sampling) from d 44 to 46 (period 1), 72 to 74 (period 2), and 100 to 102 (period 3) every 8 h for determination of total-tract apparent nutrient digestibility (**TTAD**). This sample schedule was adopted to understand if the effects on TTAD were different over time or, in other words, if differences would be observed as the BAC supplementation was extended. During the sampling period, TMR samples and orts were collected for determination of total-tract apparent nutrient digestibility according to the following equation: TTAD (%) = [(DMI × NCDM) − (FDM × NCFM) × 100]/(DMI × NCDM), where TTAD = total-tract apparent digestibility, DMI is in kilograms, NCDM = nutrient content of the DMI (%), FDM = fecal DM (kg), and NCFM = nutrient content of the fecal DM (%). The only exception was starch digestibility, which was determined according to the equation reported by Fredin et al. (2014). Fecal material (∼100 g) was weighed and stored at 18°C for subsequent laboratory analysis. Frozen samples were thawed and dried in a forced-air oven at 55°C for 96 h. The TMR, orts, and fecal samples were ground into a 1-mm screen using a Wiley mill (Marconi Equipamentos Laboratories, Piracicaba, SP, Brazil). Dry matter composition was determined by drying the samples in an oven at 105°C for 24 h and NDF content was analyzed according to procedures described by Van Soest et al. (1991) with the addition of thermostable α-amylase and sodium sulfite in an Ankom-200 (Ankom Tech Corp., Fairport, NY). The starch content of the TMR and fecal samples was determined by colorimetry as described in Fernandes et al. (2022), adapted from Hall et al. (2015).

Sixteen rumen-fistulated nonlactating multiparous Holstein cows were ranked by initial BW and assigned to the same TMR and treatment groups described in experiment (**Exp.**) 1, resulting in 8 cows/treatment group for CON and BAC, respectively. Cows were housed in individual concrete-surface pens that contained a feed bunk and water trough that ensured ad libitum intake of both TMR and water, respectively. The TMR was fed twice daily and treatments were offered for 25 d and the sampling period lasted from d 22 to 25.

Starting on d 22 of the study, rumen samples were taken at 0 (immediately before feeding), 12, 24, 48, and 72 h post-TMR feeding for determination of rumen pH, ammonia-N (NH_3_-N), and ruminal degradability. Ruminal fluid samples were collected (approximately 100 mL) by squeezing the ruminal contents into 4 layers of cheesecloth and the ruminal fluid pH was immediately determined (Digimed-M20, Digimed Instrumentação Analítica, São Paulo, SP, Brazil). Approximately 50 mL of the ruminal fluid was collected and stored (−20°C) for subsequent analysis. Frozen ruminal samples were prepared for analysis by thawing, centrifuging (15,000 × *g*) for 10 min at room temperature, and analyzing for rumen NH_3_-N according to procedures described by Broderick and Kang (1980). For ruminal degradability determination, Dacron bags (50 ± 10 μm pore size and 10 × 20 cm bag size; Ankom Technology Corp.) containing 4 g (DM basis) of ground TMR (2-mm screen, Wiley Mill, model 4, Arthur H. Thomas, Philadelphia, PA) were suspended into the ruminal ventral sac of each cow and incubated in duplicates for 0 (immediately before feeding), 12, 24, 48, and 72 h. Before ruminal incubation, all bags were soaked in warm water (39°C) for 15 min. After ruminal incubation, bags were washed repeatedly with running water until the rinse water was colorless and subsequently dried for 96 h at 50°C in a forced-air oven. The 0-h bags were not incubated in the rumen but were subjected to the same soaking, rinsing, and drying procedures applied to the ruminally incubated bags. Last, dried samples were weighed for residual DM and starch determination.

The primary endpoint of Exp. 1 was feed efficiency and sample size was determined using the University of British Columbia Power Calculator (https://www.stat.ubc.ca/∼rollin/stats/ssize/n2.html), setting an α of 0.05 and power of 0.80 to detect 3.2% difference. For both experiments, all data were analyzed with the MIXED procedure of SAS (version 9.4, SAS Institute Inc., Cary, NC), using cow as the experimental unit, the Satterthwaite approximation to determine the denominator df for the test of fixed effects, and block (Exp. 1) or cow (Exp. 2) was used as the random variable. The SCC data were log-transformed for statistical analysis and converted back to 1,000 cells/mL for reporting in the article. Variables collected over time were also evaluated with the repeated statement of SAS, using week, period, or hour as the specified repeated term and cow(treatment) in the subject for both experiments. For these variables, the covariance structure used was first-order autoregressive, which provided the smallest Akaike information criterion and hence the best fit for all variables analyzed. In Exp. 1, performance results (milk yield, DMI, feed efficiency, milk composition, and blood variables) are reported as covariately adjusted means, whereas nutrient digestibility (Exp. 1) and all data from Exp. 2 are reported as LSM, being separated using the PDIFF option of SAS. Statistical significance was set at *P* ≤ 0.05 and tendencies were denoted if 0.05 < *P* ≤ 0.10. Results are reported according to main effects if no interactions were significant or according to highest-order interaction detected.

All data were significant covariates (*P* ≤ 0.001) and did not differ among treatments (*P* ≥ 0.07; data not shown). No significant treatment × week interactions (*P* ≥ 0.06) were observed on productive data and, therefore, only main effects will be discussed here. Dry matter intake was reduced by 7.1% following BAC supplementation (*P* = 0.03), no differences were observed on milk yield (*P* = 0.77), and cows offered BAC had a greater feed efficiency (7.5%) compared with CON cohorts (*P* = 0.04; Table 2). *Bacillus* spp. supplementation increased milk fat content by 18.5% (*P* < 0.001), reduced MUN (3.8%; *P* < 0.01), tended to reduce milk protein content (*P* = 0.08), and did not affect milk lactose content or SCC (*P* ≥ 0.49; Table 2).

**Table 2:** Performance, metabolic, and digestibility results, and rumen fermentation traits of Holstein females supplemented or not (CON) with a *Bacillus*-based direct-fed microbial (BAC) in experiments 1 and 2^1^, ^2^, ^3^

Item	Treatments		SEM	*P*-value
	CON	BAC		
Experiment 1				
DMI, kg/d	15.4	14.3	0.37	0.03
Milk yield, kg/d	20.0	19.9	0.66	0.77
Feed efficiency, kg milk/kg DMI	1.33	1.43	0.038	0.04
Milk composition, %				
Fat	3.35	3.97	0.068	<0.0001
Protein	3.23	3.08	0.071	0.08
Lactose	4.13	4.11	0.030	0.56
SCC, × 1,000 cells/mL	173.0	140.6	33.58	0.49
MUN, mg/dL	13.0	12.5	0.11	<0.01
Plasma variable				
BUN, mg/dL	15.3	13.9	0.99	0.02
Glucose, mg/dL	54.5	55.8	1.10	0.03
Nutrient digestibility, %				
DM	56.2	56.2	0.54	0.91
NDF	41.2	43.3	0.90	0.01
Starch	95.5	95.5	0.21	0.82
Experiment 2				
Rumen pH	6.46	6.68	0.060	0.02
Rumen NH_3_-N, mg/dL	5.92	4.32	0.795	0.18
DM degradability, %	69.97	71.03	0.561	0.01

1 Experiment 1 consisted of 60 mid- to late-lactating Holstein cows offered a silage-based TMR period containing or not (CON; n = 30) 3 g of BAC (n = 30) for 12 wk.

2 Experiment 2 consisted of 16 rumen-fistulated nonlactating Holstein cows offered the same TMR from Exp. 1 for 25 d (n = 8 animals/treatment).

3 CON = basal TMR offered to ensure ad libitum intake throughout the trial; BAC = CON diet with the addition of 3 g/head per day of a *Bacillus*-based DFM (BOVACILLUS, Novonesis, Lyngby, Denmark).

A treatment × hour interaction was observed for plasma glucose (*P* < 0.01), as BAC cows had greater plasma glucose 1 h postfeeding (*P* < 0.0001; 55.0 vs. 58.7 mg/dL; SEM = 1.20), but no further differences were observed (*P* ≥ 0.15; data not shown). Last, mean BUN was reduced (*P* = 0.02) by 9.1% and plasma glucose was 2.3% greater (*P* = 0.03) in mid- to late-lactating cows offered BAC (Table 2). No treatment effects were observed either on DM or starch digestibility (*P* ≥ 0.82), whereas NDF digestibility was 5.0% greater for BAC versus CON cows (*P* = 0.01; Table 2).

A treatment × hour interaction was observed on DM degradability (*P* = 0.03), as the degradability was greater at 12 h postfeeding in BAC cows (*P* < 0.001; Figure 1A). Although a treatment × hour interaction was not observed on rumen pH (*P* = 0.95), BAC cows tended to have a greater rumen pH versus CON at 12, 24, and 72 h (*P* ≤ 0.09; Figure 1B). Moreover, mean rumen pH (3.4%) and DM degradability were greater (1.5%; *P* ≤ 0.02) in BAC-fed cows, but no differences were observed on rumen NH_3_-N (*P* = 0.18; Table 2).

**Figure 1:**
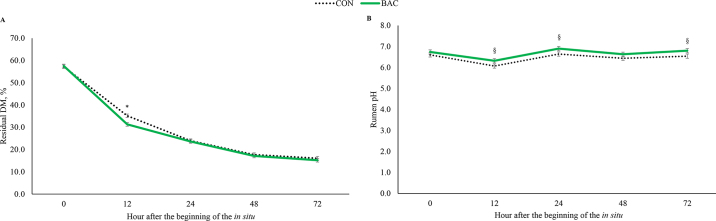
Dry matter degradability (A) and rumen pH (B) of rumen-fistulated nonlactating Holstein cows offered a silage-based TMR containing or not (CON; n = 8) 3 g/head per day of a *Bacillus*-based direct-fed microbial (BAC; n = 8; BOVACILLUS, Novonesis, Lyngby, Denmark). A treatment × day interaction was observed for DM degradability (*P* = 0.03). *Denotes significance at *P* < 0.05 level, and § denotes a tendency at 0.05 < *P* ≤ 0.10. Error bars are the SEM obtained from the statistical analysis.

The main goal of the present experiments was to evaluate the effects of supplementing a *Bacillus*-based DFM on performance of mid- to late-lactating Holstein dairy cows and on rumen fermentation traits of rumen-fistulated Holstein females offered a corn silage–based TMR. Part of the aforementioned goals were derived from the fact that the challenge of providing dairy products to an increasing population are partially dependent on the efficiency of nutrient utilization by the cows, specifically carbon and nitrogen (Asner and Archer, 2010; Galloway et al., 2010). Previous research suggested that dilution of nutrient requirements for maintenance and genetic selection for greater productivity (Bauman et al., 1985; USDA, 2013) would be enough to optimize feed efficiency, but these trends have not been confirmed over time, partially because of the loss of digestible energy associated with high passage rate in lactating dairy cows with high DMI and milk production (NASEM, 2021). Therefore, different strategies that optimize feed efficiency in dairy cows are warranted in further research efforts, as this is a key trait affecting the profitability of the dairy farm (Connor, 2015; Bach et al., 2020). Bacteria-based DFM has been shown to optimize feed efficiency in lactating dairy cows (Valldecabres et al., 2022; Nehme Marinho et al., 2024), highlighting this technology as feasible and a potential candidate to promote the profitability of dairy production settings.

Several factors, including stage of lactation, nutrient digestibility, rumen microbial population, health status, and environment, significantly affect feed efficiency in the dairy cow herd (Bach et al., 2020). During early lactation, the dairy cow uses its own body reserves to sustain milk production, and as lactation progresses, cows have a lower feed efficiency (Li et al., 2017) due to nutrient partitioning toward body reserves (Bach et al., 2020). Nutrient digestibility has been associated with improved feed efficiency and specially more in cows fed a low-starch diet (15% starch; Potts et al., 2017). Feeding a *Bacillus*-based DFM to mid- to late-lactating dairy cows resulted in a greater feed efficiency (+7.5%; Exp. 1), while also stimulating nutrient degradation and digestion (DM and NDF; Exp. 1 and 2), a more stable rumen environment (rumen pH increased by 3.4%; Exp. 2), and the modulation of metabolites associated with rumen fermentation and metabolism (glucose, BUN, and MUN). Hence, BAC demonstrated to be a reliable tool and strategy to optimize feed efficiency by decreasing DMI of late-lactating dairy cows, without affecting their productive performance. Moreover, the increased blood glucose levels might be at least one of the basic triggering effects that help us to explain the decreased DMI observed in Exp. 1, as glucose is originated from propionate fermentation in the rumen and it has been associated with the control of appetite in dairy cattle (Allen, 2000). Feedlot bulls offered a high-starch diet also had a lower DMI and greater feed efficiency when BAC was fed (Cordeiro et al., 2024). In early to mid lactation, BAC feeding has not affected DMI of dairy cows offered low- and high-starch diets (Oyebade et al., 2023; Cappellozza et al., 2024; Terré et al., 2024). However, additional studies are required to understand the effects of BAC and rumen fermentation traits, specifically VFA and microbiome profile, when offered TMR-based diets.

In agreement with our results, others have demonstrated that *B. licheniformis* 809 and *B. subtilis* 810 supplementation has improved feed efficiency in early- to mid-lactating dairy cows consuming diets containing between 14.0% and 28.5% starch (Bach et al., 2024; Cappellozza et al., 2024; Terré et al., 2024). In contrast, feeding *B. subtilis* C-3102 did not improve feed efficiency or nutrient digestibility in late-lactating dairy cows (Souza et al., 2017), suggesting that the specific strain of *B. subtilis* is important for probiotic selection and, therefore, for reaching the desired goal of the dairy production. In vitro inoculation of *B. licheniformis* 809 and *B. subtilis* 810 improved DM and NDF degradability of forage-based substrates and dairy TMR, but also starch degradability of concentrate feeds (Pan et al., 2022; Cappellozza et al., 2023), supporting our current results (in vivo and in situ) and also corroborating the enzyme-producing ability of *Bacillus* spp. (Luise et al., 2022). Moreover, the greater blood glucose concentration and milk fat corroborates with such responses. Recently, Izquierdo et al. (2024) reported that grazing beef heifers fed *B. licheniformis* 809 and *B. subtilis* 810 from mid gestation to postcalving had a greater prepartum BCS change and plasma glucose concentration when compared with nonsupplemented cohorts, suggesting that these positive changes were associated with improved forage utilization and *Bacillus* spp. enzymatic activity. Last, it is noteworthy mentioning that the stage of lactation significantly affects feed efficiency of the herd, as demonstrated by others (Connor, 2015; Bach et al., 2020). Hence, improving feed efficiency in late-lactating dairy cows could be associated with a greater rumen function, but also to a greater supportive effect on health of the herd, as late-lactating dairy cows have been reported to develop insulin resistance (Baruselli et al., 2016; Leiva et al., 2018), which is associated with inflammation in ruminants (de Sousa et al., 2022). In fact, beef steers challenged with LPS had greater concentrations of inflammatory markers, but reduced nutrient digestibility (Lippolis et al., 2017), suggesting a potential role of inflammation on rumen and gastrointestinal tract (**GIT**) health, integrity, and microbiome function (Boll et al., 2024). However, this topic needs to be properly evaluated and assessed in late-lactating dairy cows fed a *Bacillus*-based DFM.

An interesting and novel finding of the present study is that rumen-fistulated cows fed the TMR containing *B. licheniformis* 809 and *B. subtilis* 810 had a greater mean rumen pH compared with the control cows. Moreover, DM degradability was greater at 12 h in *Bacillus*-fed cows, whereas rumen pH tended to be greater in the same time point, indicating that feeding a *Bacillus*-based DFM maintained a more adequate and synchronized environment to support the activity of fibrolytic and proteolytic bacteria. Additionally, rumen pH also tended to be greater at 24 and 72 h post-TMR feeding in BAC versus CON. Supporting our findings, it has been recently demonstrated that the supplementation of *B. licheniformis* 809 and *B. subtilis* 810 altered the microbial community in dairy (Lamontagne et al., 2023) and beef (Linde et al., 2023) cattle, likely favoring rumen function, health, and thus, nutrient utilization by the microbial population of the host. Moreover, in rumen-fistulated beef cows offered a low-quality diet, BAC feeding increased rumen pH at 16 and 20 h postfeeding, while concomitantly increasing nutrient degradation and digestion in the rumen and whole GIT (Limede et al., 2024; Marques et al., 2024). Contrary to our results, Sun et al. (2013) reported a lower mean rumen pH and NDF digestibility in dairy cows fed *B. subtilis natto* during the first trimester of lactation, suggesting that the activity of cellulolytic bacteria was reduced. Nonetheless, differences in the composition and nutritional profile of the diet, as well as differences among the bacilli strains should be considered when evaluating such differences among the studies. Moreover, *Bacillus* spp. may have direct or indirect mechanisms that stimulate the cellulolytic activity in the rumen, potentially benefiting rumen function and, hence, nutrient degradation by maintaining rumen pH at more adequate levels for gram-positive bacteria. Last, the improvements in nutrient utilization and feed efficiency could be multifactorial, as supporting both the rumen and lower GIT environment can benefit milk yield, milk composition, and feed efficiency.

In summary, feeding a *Bacillus*-based DFM to mid- to late-lactating dairy cows improved feed efficiency by 7.5% and increased mean circulating concentration of glucose (2.3%), while also stimulating DM and NDF digestibility. Nonetheless, additional studies are warranted to understand the microbial community and the metabolic pathways that are altered, if any, in dairy cows offered a *Bacillus*-based DFM.
